# The Current State of Surgical Interest Amongst Rural Junior Doctors and Medical Students

**DOI:** 10.7759/cureus.102887

**Published:** 2026-02-03

**Authors:** Harry Jin, Emily Chen, Avjit Singh, Matthew Lyon

**Affiliations:** 1 General Surgery, Gold Coast University Hospital, Gold Coast, AUS; 2 General Surgery, Toowoomba Hospital, Toowoomba, AUS; 3 General Surgery, Townsville University Hospital, Townsville, AUS

**Keywords:** junior doctor training, medical student, rural areas, rural surgeon, surgical career interest

## Abstract

Background

Over recent years, there has been a declining interest in the pursuit of a surgical career by medical students and junior doctors. There is a present need to actively foster interest in surgical careers, which is especially true in the rural setting. The aim of this study was to determine the current level of interest in a surgical career amongst medical students and junior doctors and identify factors that may influence desires to pursue a surgical career.

Methods

In this cross-sectional study, a survey was distributed to medical students and junior doctors in their final week of placement on a surgical term at a major surgical referral centre in regional Australia. Questions regarding participants’ interest in a surgical career, self-reported engagement with operating theatres, subjective opinions on barriers and enablers of surgical careers were asked. Survey responses were assessed on a five-point Likert scale.

Results

A total of 69 participants completed the survey, including 30 students (43.5%) and 39 junior doctors (56.5%). As training level increased from medical student to junior doctor, interest in surgery declined (OR = 0.51, 95% CI: 0.35 - 0.71, *p *< 0.001). A perception of bad work-life balance was reported as a negatively contributing factor by 91% of participants. Engagement in the operating theatres, baseline surgical interest, and being a medical student were associated with a statistically significant positive change in surgical interest. Multivariable analysis showed that baseline surgical interest was the only predictor of positive change in surgical interest. Students and doctors who grew up in a rural setting were significantly more likely to express desires to return to a rural setting for long-term practice (Wilcoxon rank-sum test: W=389, *p *= 0.01)

Conclusion

Baseline surgical interest is a strong predictor of a positive change in surgical interest following a surgical rotation. Surgical interest appears to decline as students and junior doctors progress through their training, highlighting the necessity to instil interest at an early stage in their medical careers.

## Introduction

Over recent years, there has been a declining interest in the pursuit of a surgical career by medical students and junior doctors [1]. In the 2023 annual activities report published by the Royal Australasian College of Surgeons (RACS), there were a total of 799 applicants to surgical training [2]. This is in contrast to the 1003 applicants in 2016 and 1150 applicants in 2014. Similarly, in the United States and Canada, there has been a reduction in the proportion of medical graduates applying for surgical residency programmes [3]. In a United States-based 2005 survey, 45% of first-year medical students expressed interest in a surgical career, whereas only 7% of graduating students expressed interest [4].

This gradual decline in interest over the years can be reflected in the state of the current surgical workforce. In a 2021 population-based audit, the field of general surgery experienced a decrease in the number of practicing surgeons and an increase in patients-per-physician from 2012 to 2019 [5]. This deficit in the workforce was especially pronounced in rural settings where the predominant surgical workforce is made up of fly-in, fly-out (FIFO) visiting surgeons [6]. Evidently, there is a current need to actively foster interest in a surgical career amongst medical students and junior doctors, especially in the rural setting.

In Australia, following graduation, medical students enter the workforce as interns and progress through the years as resident medical officers (RMOs) prior to selecting a subspecialty interest, where an additional rigorous selection process is undertaken to be selected into a specialty training programme. This allows junior doctors to broaden their experiences and develop a deeper understanding of each field of medicine before committing to a chosen specialty.

The aim of this study was to determine the current level of interest amongst medical students and RMOs in a surgical career and identify factors that may influence desires to pursue a surgical career. The primary outcome was the degree of surgical interest amongst medical students and junior doctors at the conclusion of their surgical term. Secondary outcomes included self-reported negatively and positively influencing factors that participants experienced during their term that changed their perception of a surgical career.

## Materials and methods

Design

This was a cross-sectional survey-based observational study conducted at a single surgical referral centre, Toowoomba Hospital, in Toowoomba City, Queensland, Australia. The primary outcome was the subjective change in desire of participants in pursuing a surgical career following the completion of their surgical term. Secondary outcomes included participants’ self-reported desire to pursue a career with technical elements, their baseline level of surgical interest, a desire to practice in a rural/regional setting, and self-perceived barriers and enablers of pursuing a surgical career. Ethical approval was obtained through the local Human Research Ethics Committee (reference number: HREC/2024/QTDD/95652).

Participants

Medical students and RMOs rotating through our hospital’s general surgical department were invited to participate in this study. At our institution, medical students rotate through various surgical specialties (General Surgery, Otolaryngology, Orthopaedics, and Urology) for a total of six weeks, and junior doctors rotate through 10-week terms of various specialties, of which general surgery, otolaryngology, orthopaedics, and urology are options. Inclusion criteria consisted of completion of a general surgical term either as a medical student in their clerkship years or as an RMO. An “RMO” was defined as a medical doctor not currently engaged in a specialty advanced training pathway. International medical graduates were also included in the study. Doctors who were in an advanced training pathway in any medical or surgical specialty were excluded from the survey.

Survey distribution

Surveys were conducted electronically through SurveyMonkey (SurveyMonkey Inc., San Mateo, California, United States). Surveys were distributed through emails, flyers containing a link to the survey, as well as word of mouth during the 2023-2024 clinical year. The survey was distributed in the final week of each participant’s term in a general surgery unit. Study investigators did not physically approach individual participants to avoid the potential for bias in the responses. Only de-identified data was collected through the survey.

Statistical analysis

Statistical analysis was performed using R version 4.3.1, 2023-06-16 (R Foundation for Statistical Computing, Vienna, Austria, https://www.R-project.org/). Frequency tables were used to present descriptive statistics. The Mann-Whitney U test was used to compare the Likert-scale outcome variables between independent groups. Fisher’s exact test was used to evaluate the associations between categorical variables. An ordinal logistic regression model was used to compare Likert-scale outcomes while adjusting for confounders. A p-value of less than 0.05 was considered statistically significant.

## Results

A total of 69 participants completed the questionnaire and were included in the analysis with a response rate of 88%. Of the surveyed participants, 30 (43.5%) were medical students, and 28 (40.6%) were males. Basic demographic data is displayed in Table 1. All participating medical students were in their clinical years of medical school. Twenty-two (31.9%) participants were post-graduate year (PGY) 1, 11 (15.9%) were PGY-2, and six (8.7%) were PGY-3. Thirty-two (46.4%) participants indicated they had grown up in a rural setting. More medical students were female, were younger, and were more likely to have grown up in a rural environment. Medical students were more likely to be interested in a surgical career than their RMO counterparts (Wilcoxon rank-sum test, p < 0.001). Following completion of their surgical terms, medical students were more likely to report a positive change in their desire to pursue a surgical career (Wilcoxon rank-sum test, p < 0.001).

**Table 1:** Participant characteristics Wilcoxon rank-sum test and Chi-squared test performed between RMO/Intern and Student groups ^Assessed on a 1-5 Likert scale: 1 – Strongly negative, 3 – Neutral, 5 – Strongly positive RMO: resident medical officer; IQR: interquartile range

Parameters	RMO/Intern (n=39)	Student (n=30)	Test statistic	p
Male/Female	20/19	8/22	χ² = 4.26	0.039
Age (years), mean±SD	27 ± 2.1	24 ± 2.6	W = 992.5	< 0.001
Rural upbringing, n (%)	14 (36%)	18 (60%)	χ² = 3.96	0.047
Interested in a surgical career, median (IQR)^	2 (1-2)	3 (2-4)	W = 271.5	< 0.001
Interested in a career with technical elements, median (IQR)^	4 (4-5)	4 (4-5)	W = 573	ns
Interested in a rural career, median (IQR)^	3 (2-4)	3.5 (3-4)	W = 271.5	ns
Changes in desire to pursue surgery after completing term, median (IQR)^	3 (2-3.5)	4 (3.25-4)	W = 334.5	< 0.001

**Figure 1:**
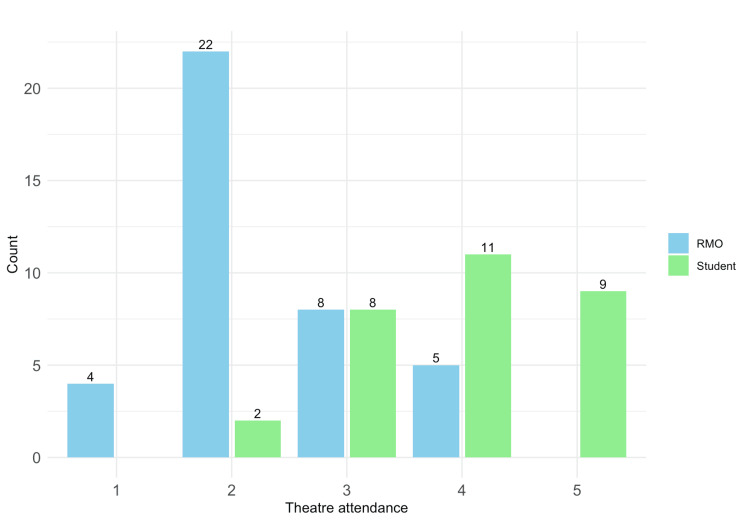
Participation in operating theatres Data presented as n RMO: resident medical officer

Self-reported degree of participation in the operating theatres was also higher in medical students (Wilcoxon rank-sum test, p < 0.001) (Figure 1). 

Self-reported positive and negative motivating factors impacting surgical interest are outlined in Table 2. Of note, only 6% of participants reported a good work-life balance as a positive motivating factor, while most participants cited a poor work-life balance (91%) as a negative motivating factor. Of the responses, only the feeling of being disengaged with the team as a negative motivating factor was reported at a higher rate by medical students compared to RMOs (8% vs 27%, p = 0.047). Other reported positive factors included free food during seminars and the satisfaction of learning new skills. Other reported negative factors included bullying and rigorous research requirements for admission to training.

**Table 2:** Subjective experiences that influenced interest in surgery during the term Fisher’s exact test performed between RMO/Intern and student groups * free food during seminars and the satisfaction of learning new skills.; ** bullying and rigorous research requirements for admission to training RMO: resident medical officer

Parameters	RMO/Interns	Students	Total	p
	(n=39)	(n=30)	(n=69)	
Positive influence				
Positive role models	22 (56%)	23 (77%)	45 (65%)	ns
Good work-life balance	1 (3%)	3 (10%)	4 (6%)	ns
Feeling like you were a part of the team	21 (54%)	23 (77%)	44 (64%)	ns
Enjoyed clinical elements of the job	22 (56%)	21 (70%)	43 (62%)	ns
Others*	2 (5%)	0 (0%)	2 (3%)	ns
Negative influence				
Negative role models	10 (26%)	11 (37%)	21 (30%)	ns
Bad work-life balance	35 (90%)	28 (93%)	63 (91%)	ns
Feeling like you were not a part of the team	3 (8%)	8 (27%)	11 (16%)	0.047
Did not enjoy clinical elements of the job	6 (15%)	4 (13%)	10 (14%)	ns
Others**	0 (0%)	2 (6%)	2 (3%)	ns

Baseline level of surgical interest was negatively correlated with increasing level of training (OR = 0.51, 95% CI: 0.35 - 0.71, p < 0.001), and participants who were interested in incorporating technical elements in their career were also more likely to be interested in a career in surgery (OR = 2.88, 95% CI: 1.68 - 5.33, p < 0.001).

The most significant predictor of a positive change to surgical interest was the participants’ baseline level of surgical interest (Table 3). On univariate analysis, degree of participation in theatres, being a medical student, earlier career stage, and baseline interest in a career incorporating technical elements were also predictors of a positive change in surgical interest. On multivariate analysis, only the baseline level of surgical interest remained statistically significant (Table 4).

**Table 3:** Ordinal logistic regression examining predictors of change in surgical interest following completion of a surgical term

Predictor	OR	95% CI	z	p
Degree of theatre participation	2.09	1.37 – 3.32	3.283	0.001
Being a medical student	5.08	1.95 – 14.23	3.227	0.001
Baseline surgical interest	4.98	2.89 – 9.43	5.363	< 0.001
Baseline interest in technical career	1.74	1.09 – 2.86	2.298	0.021
Level of medical education	0.58	0.40 – 0.81	-3.129	0.002
Male gender	0.49	0.20 – 1.18	-1.574	ns
Age	1	0.86 – 1.17	0.066	ns

**Table 4:** Multivariable analysis examining predictors of change in surgical interest following completion of a surgical term

Predictor	OR	95% CI	z	p
Degree of theatre participation	0.99	0.53 – 1.88	-0.037	ns
Being a medical student	0.8	0.06 – 9.92	-0.170	ns
Baseline surgical interest	4.9	2.53 – 10.49	4.414	< 0.001
Baseline interest in technical career	0.93	0.50 – 1.73	-0.230	ns
Level of medical education	0.85	0.37 – 1.89	-0.389	ns

Four (12.5%) out of the 32 participants who reported having grown up in a rural setting reported they were interested in a surgical career compared with nine (26%) out of 35 participants who were not of rural origin (Wilcoxon rank-sum test, p = 0.225). Participants who were of rural origin were significantly more likely to have a desire to practice rurally at the end of their training (Wilcoxon rank-sum test, p = 0.01).

## Discussion

In Australia, the total number of applicants to surgical training is declining [2]. This phenomenon can be seen worldwide and may represent a widespread change in the perception of the surgical career [1,7]. What was once seen as a prestigious, highly desirable vocation may now be perceived as an occupation with poor work-life balance and a rigorously competitive admission standard. Nonetheless, admission into surgical training and residency programs remains highly competitive, with competition ratios of up to 27 to 1 for certain surgical subspecialties [8]. While it is likely that there will always be more applicants than there are training positions, the total number of applicants may continue to fall. This may give rise to a scenario where pre-training junior surgical roles, which constitute a significant portion of surgical service provision, may go unfilled [9].

Our study demonstrates that most medical students and junior doctors perceive a surgical career as having poor work-life balance. In Australia, this is compounded by the fact that surgical trainees are required to relocate significant geographic distances several times throughout their training, often uprooting their families to do so. Additionally, admission into surgical training is a rigorous exercise often necessitating significant time, resources, and energy out of usual working hours to complete research projects, attend workshops, and present abstracts at national and international conferences [10, 11]. In a 2015 study conducted in the United Kingdom, the average amount spent for applicants to specialty training (excluding compulsory expenses) was £5220 [12]. In higher competitive specialties such as otolaryngology, the financial cost of a residency match is reported as high as $20,000 USD [13]. This will often be in addition to debt accrued over the course of the primary medical degree. The prospect of taking on such a financial burden may deter students and junior doctors to ever consider a surgical career in the first place. Over recent years, there has been a paradigm shift in medical workplace legislation and culture that aims to rectify this perception [14,15]. Nonetheless, continued efforts should be made to mitigate this perception of a “poor work-life balance” amongst medical students and junior doctors.

Factors that may positively influence medical students’ and junior doctors’ interest in a surgical career include pre-clerkship exposure, degree of clinical exposure, presence of a positive role model, self-perceived level of engagement with the surgical team, and engagement in technical skills workshops [16-20]. Our study found that most participants enjoyed the clinical aspects of surgery, and a desire to incorporate technical elements into their careers was almost universally observed. It has been well documented that “hands-on” technical workshops foster surgical interest in medical students [21,22]. Consistent with this finding, our study demonstrates that participants who attended theatres more frequently were more likely to have a positive change in their attitude towards a surgical career. Structured and allocated theatre sessions may foster surgical interest in students and junior doctors alike, and engagement in the operating theatres should be actively encouraged. This is especially true for RMOs, as our study demonstrated they were far less likely to have opportunities to attend operating theatres.

Unsurprisingly, participants who had a high level of baseline surgical interest were more likely to have a positive experience and end the rotation with an increased desire to pursue surgery. This was statistically significant in multivariate analysis. We also found that as participants progressed through stages of medical training, interest levels were likely to decline. This highlights the need to engage medical students early in their training to foster longitudinal interest in surgery. An important factor we encountered was the potential negative influence of the feeling of not being “part of the team” by medical students. This is important to recognise, as it is something that can be remedied by introducing strategies in teaching hospitals. For example, allocating specific responsibilities to medical students such that they can feel a part of the team, contributing in a meaningful way to patient care.

Our study also demonstrated that students and doctors who were of rural origin were more likely to express desires of returning to practice rurally following completion of their training. This is consistent with reported literature that indicates medical students with a rural background are more likely to have intent for future rural practice [7,23]. Our study also demonstrated no difference in surgical interest between those who were of rural origin and those who were not. This highlights the necessity to actively foster surgical interest amongst medical students and junior doctors with rural backgrounds to help bolster the rural surgical workforce in the future.

Limitations

This was a cross-sectional survey that did not take into consideration the changing interests of junior medical staff as they progressed through their formative years in the workforce. Recency bias of having just completed the surgical term may have influenced the participants’ responses in a more positive or more negative light. Future studies should focus on following participants as they progress through their medical training to see how external factors may positively or negatively influence surgical interest over time.

Another limitation of this study is that it was conducted in a single regional/rural centre. Over recent years, there has been a strong shift in Australia to fostering interest and incentivising junior medical staff to pursue a more “generalist” rural pathway. As such, our institution may select students and junior doctors who are more interested in pursuing these generalist pathways rather than specialty areas and therefore underrepresent the degree of surgical interest overall, especially compared to metropolitan centres. Nonetheless, this study provides important insights into surgical interest amongst rural medical students and doctors who are more likely practice rurally.

## Conclusions

Baseline surgical interest is a strong predictor of a positive change in surgical interest following a surgical rotation. Surgical interest appears to decline as students and junior doctors progress through their training highlighting the necessity to instil interest at an early stage of medical education. Strategies to mitigate negative perceptions of surgery, as well as strategies to increase engagement during clinical placements are critical to longitudinally foster surgical interest in students and junior doctors.

## Appendices

Participant survey

What is your gender?

Female

Male

Prefer not to say

Other (specify)

What is your age?

[Free Text]

Do you have children?

Yes

No

Prefer not to say

Medical School year / PGY level

MD1

MD2

MD3

MD4

PGY Level: [Free Text]

Currently, what specialty are you interested in pursuing a career in?

[Free Text]

Did you grow up in a rural or regional setting?

Yes

No

Please list any surgical terms you have completed (if applicable)

[Free Text]

How interested are you in pursuing a career in surgery?

Essentially ruled out a surgical career

Not very interested in a surgical career

I'm in the middle

Leaning towards a surgical career

I am certain I want a surgical career

How interested are you in pursuing a career that incorporates technical skills?

I do not want any technical element in my career

Prefer not to have technical elements, but I would not mind

I don't mind either way

I want at least some technical element in my career

I am certain I want a career that incorporates technical elements

How interested are you in working in a rural/regional setting in the long term?

I definitely do not want to work in a rural setting

I would prefer not to work in a rural setting

I would not mind either way

I would prefer to work in a rural setting

I definitely want to work in a rural setting

Have you completed a surgical term?

Yes

No

How often did you scrub in to operations during your term?

Never

Rarely - Only a few times during my term

Sometimes - Every now and then

Often - At least once or twice a week

Very often - I took every opportunity to scrub

Are there any factors during the surgical rotation that impacted on your interest in pursuing a surgical career in a positive way? (choose as many)

Positive role models

Good work-life balance

Feeling like you were a part of the team

Enjoyed the clinical elements of the job (operating, types of pathologies, caring for patients on the ward, etc)

Other (please specify) [Free Text]

Are there any factors during the surgical rotation that impacted on your interest in pursuing a surgical career in a negative way? (choose as many)

Negative role models

Bad work-life balance

Not feeling like you were a part of the team

Did not enjoy the clinical elements of the job (operating, types of pathologies, caring for patients on the ward, etc.)

Other (please specify) [Free Text]

How did completing your surgical term aﬀect your desire to pursue a career in surgery?

It put me oﬀ surgery completely

It reduced my desire to pursue a surgical career

Had no impact on my career decision

It increased my desire to pursue a surgical career

It made me want to become a surgeon

What do you wish you got to do more during your surgical term?

[Free Text]
